# Complete Genome Sequence of a Veterinary Pseudomonas aeruginosa Isolate, Pa12

**DOI:** 10.1128/MRA.00398-21

**Published:** 2021-07-01

**Authors:** Keisuke Nakamura, Jumpei Fujiki, Takaaki Furusawa, Tomohiro Nakamura, Satoshi Gondaira, Michihito Sasaki, Masaru Usui, Hidetoshi Higuchi, Hirofumi Sawa, Yutaka Tamura, Hidetomo Iwano

**Affiliations:** a Laboratory of Veterinary Biochemistry, Rakuno Gakuen University School of Veterinary Medicine, Ebetsu, Japan; b Laboratory of Animal Health, Rakuno Gakuen University School of Veterinary Medicine, Ebetsu, Japan; c Division of Molecular Pathobiology, International Institute for Zoonosis Control, Hokkaido University, Sapporo, Japan; d Laboratory of Food Microbiology and Food Safety, Rakuno Gakuen University School of Veterinary Medicine, Ebetsu, Japan; e International Collaboration Unit, International Institute for Zoonosis Control, Hokkaido University, Sapporo, Japan; f Center for Veterinary Drug Development, Rakuno Gakuen University, Ebetsu, Japan; Indiana University, Bloomington

## Abstract

Pseudomonas aeruginosa causes various opportunistic infections in animals. Here, we report the complete genome sequence of P. aeruginosa strain Pa12, a fluoroquinolone-resistant isolate from a canine skin lesion. To expand the molecular antimicrobial characteristics of the isolate, the whole Pa12 genome was sequenced and assembled via long- and short-read platforms.

## ANNOUNCEMENT

Pseudomonas aeruginosa is an opportunistic Gram-negative bacterium and an important pathogen of humans and animals. In veterinary medicine, P. aeruginosa typically causes skin and urinary tract infections in dogs (1–3). The recent spread of antimicrobial resistance (AMR) has become a major problem in veterinary medicine; notably, the rate of resistance to fluoroquinolones has increased rapidly (4–6).

Pseudomonas aeruginosa exhibits AMR via multiple mechanisms. Multidrug efflux (Mex) systems are associated with intrinsic fluoroquinolone resistance, and P. aeruginosa can also acquire fluoroquinolone resistance through mutations in genes of the quinolone resistance–determining region (QRDR) (7–10).

We previously isolated P. aeruginosa strain Pa12 from a canine skin lesion at a veterinary hospital in Japan (11). The specimen was cultured on sheep blood agar and MacConkey agar at 35°C for 24 to 48 h under aerobic conditions. This strain exhibited intermediate resistance to orbifloxacin in MIC assays (11, 12). In the present study, we report the complete genome sequence of P. aeruginosa strain Pa12 to enhance molecular understanding of the isolate with respect to AMR.

DNA was extracted from an overnight culture (grown at 37°C in LB medium) using phenol and chloroform (13) and purified using a QIAamp DNA minikit column (Qiagen, Hilden, Germany). The DNA concentration was determined using a Qubit fluorometer (Invitrogen, Carlsbad, CA, USA). Whole-genome sequencing was performed using MiSeq (Illumina, San Diego, CA, USA) and GridION X5 (Oxford Nanopore Technologies [ONT], Oxford, UK) platforms. Libraries for Illumina sequencing were prepared using a Nextera XT DNA library preparation kit (Illumina). The whole genome was then subjected to 300-bp paired-end sequencing on the MiSeq platform. The resulting 8,118,954 reads were trimmed of adaptors and low-quality bases (Q scores of <20), and short reads (<127 bp) were removed using Sickle v1.33. Libraries for GridION X5 sequencing were prepared using a native barcoding expansion kit (ONT) and a ligation sequence kit (SQK-LSK109; ONT) without DNA shearing. The resulting sample was sequenced on the GridION X5 platform using an R9.4.1 flow cell (ONT) and Guppy v3.2.10 for live base calling. The resulting 56,776 reads were trimmed and quality filtered using Porechop v0.2.3 and Filtlong v0.2.0 (minimum length, 1,000 bp; *N*_50_, 25,214 bp). Hybrid *de novo* assembly, genome circularization, and genome rotation were performed using Unicycler v0.4.7 (14), resulting in one circular contig with a total length of 6,411,763 bp and a G+C content of 66.4%. Finally, the assembled contig was annotated using DFAST v1.1.0 (15). Default parameters were used for all software except where otherwise noted.

The genome of Pa12 consists of one circular chromosome with a total of 5,855 coding sequences (CDSs), 12 rRNAs, and 73 tRNAs (Table 1). ResFinder v4.1 analysis (16, 17) detected five AMR genes in the Pa12 chromosome, and four Mex systems (MexAB, MexCD, MexEF, and MexXY) were identified in DFAST annotation, although no previously reported mutations were found in the Pa12 QRDR (5, 6, 10).

**TABLE 1:** Characteristics of the annotated P. aeruginosa Pa12 genome

Parameter	Finding
Component	Chromosome
Name	Pa12 chromosome
Length (bp)	6,411,763
G+C content (%)	66.4
No. of CDSs	5,855
No. of rRNAs	12
No. of tRNAs	73
AMR genes	*aph(3′)-IIb*, *catB7*, *bla*_OXA-488_, *bla*_PAO_, *fosA*

These results suggest that Pa12 fluoroquinolone resistance does not involve QRDR mutations. Therefore, other mechanisms, such as production of Mex systems, might contribute to the strain’s multidrug resistance.

### Data availability.

The complete genome sequence of P. aeruginosa Pa12 was deposited in DDBJ/ENA/GenBank under accession number AP024513. Illumina and GridION X5 sequence reads for the strain were deposited in the Sequence Read Archive (SRA) database under accession numbers DRR276530 and DRR276531, respectively.
